# A Prospective Multicenter Evaluation of the Accuracy of a Novel Implanted Continuous Glucose Sensor: PRECISE II

**DOI:** 10.1089/dia.2017.0142

**Published:** 2018-03-01

**Authors:** Mark P. Christiansen, Leslie J. Klaff, Ronald Brazg, Anna R. Chang, Carol J. Levy, David Lam, Douglas S. Denham, George Atiee, Bruce W. Bode, Steven J. Walters, Lynne Kelley, Timothy S. Bailey

**Affiliations:** ^1^ Diablo Clinical Research, Walnut Creek, California.; ^2^ Rainier Clinical Research Center, Inc., Renton, Washington.; ^3^ John Muir Physician Network Clinical Research Center, Concord, California.; ^4^ Department of Medicine, Mount Sinai Diabetes Center, New York, New York.; ^5^ Clinical Trials of Texas, Inc., San Antonio, Texas.; ^6^ Worldwide Clinical Trials, San Antonio, Texas.; ^7^ Atlanta Diabetes Associates, Atlanta, Georgia.; ^8^ Clinical Sciences and Medical Affairs, Senseonics, Inc., Germantown, Maryland.; ^9^ AMCR Institute, Inc., Escondido, California.

**Keywords:** Type 1 diabetes, Type 2 diabetes, Continuous glucose monitoring, Implantable, Accuracy, Longevity

## Abstract

***Background:*** Persistent use of real-time continuous glucose monitoring (CGM) improves diabetes control in individuals with type 1 diabetes (T1D) and type 2 diabetes (T2D).

***Methods:*** PRECISE II was a nonrandomized, blinded, prospective, single-arm, multicenter study that evaluated the accuracy and safety of the implantable Eversense CGM system among adult participants with T1D and T2D (NCT02647905). The primary endpoint was the mean absolute relative difference (MARD) between paired Eversense and Yellow Springs Instrument (YSI) reference measurements through 90 days postinsertion for reference glucose values from 40 to 400 mg/dL. Additional endpoints included Clarke Error Grid analysis and sensor longevity. The primary safety endpoint was the incidence of device-related or sensor insertion/removal procedure-related serious adverse events (SAEs) through 90 days postinsertion.

***Results:*** Ninety participants received the CGM system. The overall MARD value against reference glucose values was 8.8% (95% confidence interval: 8.1%–9.3%), which was significantly lower than the prespecified 20% performance goal for accuracy (*P* < 0.0001). Ninety-three percent of CGM values were within 20/20% of reference values over the total glucose range of 40–400 mg/dL. Clarke Error Grid analysis showed 99.3% of samples in the clinically acceptable error zones A (92.8%) and B (6.5%). Ninety-one percent of sensors were functional through day 90. One related SAE (1.1%) occurred during the study for removal of a sensor.

***Conclusions:*** The PRECISE II trial demonstrated that the Eversense CGM system provided accurate glucose readings through the intended 90-day sensor life with a favorable safety profile.

## Introduction

Numerous studies have shown that use of real-time continuous glucose monitoring (CGM) improves glycemic control^1–6^ and quality of life^7^ in individuals with type 1 diabetes (T1D) and type 2 diabetes (T2D).^8^ The clinical benefits of CGM technology are maximized in patients who regularly wear their CGM devices for at least 6 days per week^1–3,5,6,9,10^; however, many patients struggle to achieve consistent adherence.^9,10^ Moreover, many individuals who initiate CGM frequently discontinue its use. A recent survey of individuals who initiated CGM in the T1D Exchange registry found that 41% of patients discontinued use during the first year.^11^ Common reasons cited for discontinuation included discomfort wearing the sensor (42%), trouble inserting the sensor (33%), problems with adhesive securing sensor to skin (30%), challenges with the device working properly (28%), skin reactions to the sensor adhesive (18%), and interference with playing sports and activities (18%).

A novel implantable subcutaneous CGM system (Eversense CGM system; Senseonics, Inc., Germantown, MD)^12–14^ was designed to address several of the limitations of use of current CGM systems. The Eversense sensor is designed to operate for 90 days, which is intended to reduce the inconvenience and discomfort of weekly sensor insertions. The Eversense smart transmitter is worn over the sensor and wirelessly powers it to initiate the glucose measurement and the transfer of data to a Mobile Medical Application (MMA). The transmitter can be removed at any time without the need for sensor replacement, allowing greater convenience and lifestyle flexibility. In addition, hypoglycemic and hyperglycemic alerts and notifications are provided on a mobile device as well as on on-body vibratory alerts from the transmitter even when the mobile device is not nearby.

The Eversense CGM system has been evaluated in two prior published studies. A feasibility study of 12 participants with T1D found no evidence of nighttime sensor attenuation,^15^ which has been reported in transcutaneous sensors.^16,17^ The system was also evaluated in the multicenter European 180-day PRECISE pivotal study, which demonstrated that use of the CGM device provided improved glycemic control in 71 participants with T1D or T2D. The PRECISE study also established the accuracy against reference venous glucose values 40–400 mg/dL with a mean absolute relative difference (MARD) of 11.6%,^18^ which is comparable with other commercially available CGM systems.^19^ The data from the PRECISE trial were used as a training set to further improve the glucose calibration and calculation algorithm used within the system. Specifically, the algorithm was modified to improve the finger-stick calibration methodology and to account for differential physiological lag considerations.

In this report, we present findings from PRECISE II, a multicenter U.S. pivotal trial that evaluated the accuracy and safety of an updated Eversense system, which included a modified algorithm and a new sensor configuration, in individuals with T1D and T2D.

## Methods

### Study design and participants

PRECISE II was a 90-day, nonrandomized, prospective, blinded, single-arm multicenter study of the Eversense CGM system among adult participants with T1D and T2D. The study was conducted from January 2016 to July of 2016 at eight sites in the United States. The study enrolled individuals who were ≥18 years and had a clinically confirmed diagnosis of T1D or T2D for at least 1 year. Individuals were excluded from participation if they had any of the following: a history of severe hypoglycemia or diabetic ketoacidosis, requiring an emergency room visit or hospitalization during the previous 6 months; a condition preventing or complicating sensor placement, operation, or removal; symptomatic coronary artery disease, unstable angina, myocardial infarction, or stroke in the past 6 months before study; uncontrolled hypertension; hematocrit <30% or >50%; lactation, pregnancy, or intending to become pregnant during the course of the study; presence of other active implanted devices; or a condition likely to require magnetic resonance imaging for the duration of the study.

The study was performed in accordance with the Declaration of Helsinki and was approved by a centralized internal review board. Written and verbal informed consent was obtained from all participants.

### Study device

The CGM system consists of an implantable, fluorescence-based, cylindrical glucose sensor (3.5 × 18.3 mm); a smart transmitter; and a MMA that displays glucose information and operates on a mobile device, which allows users to review current and historical glucose data in real time.

**FIG. 1.**
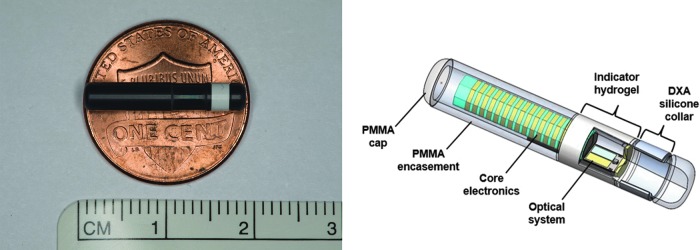
Eversense sensor. CM, centimeter; DXA, dexamethasone acetate; PMMA, polymethyl methacrylate.

The sensor contains core electronics and optics that are sealed in epoxy within a polymethyl methacrylate (PMMA) encasement (Fig. 1). The sensor is activated to measure interstitial fluid glucose every 5 min when it receives radio frequency power from the transmitter. A 100-μm thick copolymer matrix is grafted to the outside of the PMMA encasement. This embedded polymer, the indicator hydrogel, is fluorescent and uses selective, fully reversible binding between glucose and the covalently attached molecular complex to detect changes in glucose concentrations. Glucose binding results in an increase in fluorescence intensity, which is measured by the sensor's optical system. The optical system contained within the PMMA encasement includes an LED and two photodiodes, powered by a ferrite antenna substrate. These act as a miniaturized spectrofluorometer to measure the generated fluorescent intensity. The encoded data are sent to the transmitter and this information is then used to produce a glucose reading and to check the integrity of the system. The sensor has a silicone collar impregnated with a small amount (1.75 mg) of dexamethasone acetate that elutes an average of 3 μg per day over the life of the sensor to attenuate the body's local inflammatory response and prolong the sensor life. The system is designed to provide a sensor replacement alert to the user when it reaches insufficient sensitivity to glucose due to oxidative degradation of the glucose recognition chemistry.^20^ This design element is included to maintain the high degree of system accuracy throughout the entire sensor life.

The battery-powered transmitter (1.5 by 1.7 inches; 0.6 inches thick) is worn externally over the sensor and transfers glucose data to the MMA every 5 min through a secured low-energy bluetooth transmission. The transmitter also provides on-body vibrations that alert users of immediate and impending hypoglycemia and hyperglycemia. The transmitter is rechargeable and takes ∼15 min about every other day to fully charge.

### Procedures

The study consisted of seven clinic visits: a screening visit, a sensor insertion visit, four accuracy assessment visits (days 1, 30, 60, and 90), and a postsensor removal follow-up visit.

During the screening visit, participant demographic data and medical history were recorded and laboratory measurements (i.e., hemoglobin A1c [HbA1c], hematocrit, and plasma dexamethasone), a physical examination, and an electrocardiogram were performed. Female participants also had urine pregnancy testing. Sensor insertion sites, adverse events, hematocrit levels, pregnancy, and changes in medications and insulin therapy were assessed and findings recorded at all subsequent visits. HbA1c and plasma dexamethasone levels were assessed at the day 90 visit also.

**FIG. 2.**
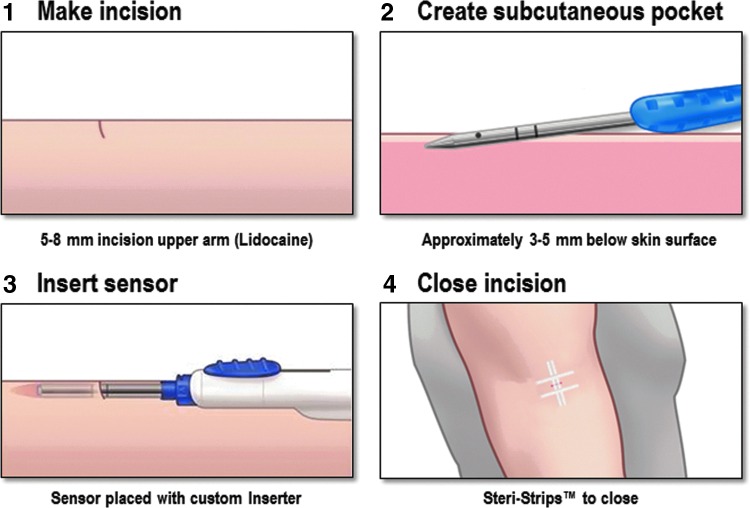
Eversense sensor insertion procedure.

Endocrinology specialists without surgical training (i.e., physicians, nurse practitioners, and physician assistants) inserted all sensors in the upper arm at the sensor insertion visit (day 0). The sensor insertion procedure is shown schematically in Figure 2. Most participants had one sensor inserted. A prespecified subset of 15 participants had bilateral sensors placed to assess intrapatient variability and the effect of compression of the system such as would occur during sleep.

After insertion, the sensor requires a 24-h warm-up phase after which the participant is prompted to begin calibration. The study-issued self-monitoring blood glucose (SMBG) meter and test strips used for calibrations were CONTOUR^®^ NEXT USB and CONTOUR NEXT blood glucose test strips (Ascensia Diabetes Care, Parsippany, NJ). Participants were asked to wear the transmitter(s) over the sensor(s) and to perform calibration twice daily. Data from the SMBG meter were collected at each follow-up visit. The CGM values and all glucose-related alerts were blinded to both the participants and investigators for the duration of the study. All diabetes care decisions were based on blood glucose meter values and clinical standards of care.

The accuracy of the system was evaluated during clinic visits on days 1, 30, 60, and 90 by comparing the glucose values measured by the CGM with those measured by a bedside glucose analyzer (2300 Stat Plus Glucose and Lactate Analyzer; Yellow Springs Instruments [YSI], Yellow Springs, OH). Qualifying participants (i.e., individuals on insulin and without gastroparesis) underwent hyperglycemia and hypoglycemia challenges on days 30, 60, and 90. The intent of the challenges was to safely manipulate the participant's blood glucose level using fasting and insulin dosing or meals of known carbohydrate content so that sensor performance could be evaluated over a wider range than might otherwise be observed. Other than during glucose challenges, participants were allowed to eat, drink, and continue their diabetes treatment regimen throughout the visits. The effect of compression was evaluated in participants with bilateral sensors by having the participant lie on one side for a period of 30 min. The effect of exercise was evaluated in participants with a single sensor who performed 30 min of upper arm exercises with barbells using the arm with the inserted sensor.

Blood samples were drawn between 7 am and 9 pm for a 4.5-h period on day 1 (venous reference measurements began after the second calibration was entered when CGM values are first available) and for a 12.5-h period on days 30, 60, and 90. Blood samples were drawn every 5–15 min, depending on the participant's blood glucose level (every 15 min for blood glucose ≥75 mg/dL, every 5 min for blood glucose <75 mg/dL). Samples were drawn every 5 min during the periods of arm exercise and compression. Each YSI blood glucose measurement was paired to the corresponding CGM measurement obtained within 5 min of the blood draw.

After the accuracy assessment at the day 90 clinic visit, venous blood samples were obtained for HbA1c and dexamethasone levels, and the sensors were removed. Ten days after removal (day 100), participants returned for follow-up and the insertion site was inspected.

### Outcome measures

The primary effectiveness endpoint was the MARD for paired sensor and YSI reference glucose measurements collected during the clinic visits through 90 days postinsertion across a glucose range of 40–400 mg/dL. Additional endpoints included Clarke Error Grid analysis^21^; sensor longevity; accuracy of rate of change (ROC); accuracy during compression and exercise; accuracy during hypoglycemic, euglycemic, and hyperglycemic states; accuracy by study visit; transmitter wear time; and alert performance. Accuracy of ROC was calculated based on linear regression of CGM in the past 15 min.

The accuracy of the CGM system to provide alerts to hypoglycemic (<70 mg/dL) and hyperglycemic (>180 mg/dL) events was determined using several measures in a retrospective manner since participants were blinded to alerts throughout the study. The “true alert rate” was calculated as the number of device alerts confirmed by YSI (the glucose level had to meet the definition of hypoglycemia or hyperglycemia within 15 min before or after an alert) divided by the number of total device alerts. The “false alert rate” was calculated as the number of device alerts not confirmed by YSI divided by the number of total device alerts. The “confirmed event detection rate” was calculated as the number of hypoglycemic or hyperglycemic events per YSI measurement that were detected by the device divided by the total number of hypoglycemic or hyperglycemic events. The “missed event detection rate” was calculated as the number of hypoglycemic or hyperglycemic events per YSI measurement that were not detected by the device divided by the number of hypoglycemic or hyperglycemic events.

The primary safety endpoint was the incidence of device-related or sensor insertion/removal procedure-related serious adverse events (SAEs) through 10 days postremoval. An independent medical monitor adjudicated all reported adverse events for relatedness to the device, sensor insertion/removal procedure, and study procedure (e.g., hyperglycemia and hypoglycemia challenges).

### Statistical methods

The prespecified analysis population for the primary effectiveness endpoint and additional endpoints was based on all evaluable glucose data from all participants with at least one paired glucose reading excluding training participants (the first participant at each site). A post hoc exploratory analysis was based on all evaluable data with at least one paired glucose reading including training participants. The safety analysis population included all participants who had a sensor placed.

The primary effectiveness endpoint was evaluated against a prespecified 20% performance goal using a generalized estimating equation with an exchangeable working correlation structure. Sensor longevity was evaluated using Kaplan–Meier analysis. A concordance analysis of the ROC in CGM and in YSI glucose was performed with each type of ROC grouped into five categories of glucose change [mg/(dL·min)]. All other effectiveness analyses were evaluated using descriptive statistics.

The proportion of participants experiencing at least one device-related or insertion/removal procedure-related SAE over the operating life of the sensor was determined along with the associated 95% confidence interval (CI).

## Results

Ninety participants (*n* = 75 single sensor and *n* = 15 bilateral sensors) were inserted with the sensor and are included in the primary effectiveness and safety populations. One hundred six sensors were placed in the study (75 single sensor participants, 15 bilateral dual sensor participants, and 1 participant who received a replacement sensor due to a suspected technical device failure). The first participant at each clinical site (*n* = 8) was considered a training participant. Eighty-two participants (91%) completed the study with day 90 data collection. Five participants experienced a sensor replacement alert before day 90, which ended glucose data collection. Two participants withdrew consent (one for being unable to tolerate intravenous access for in-clinic accuracy testing and one for scheduling difficulties). One participant was lost to follow-up; after completion of the study, the participant was located and the sensor was removed. Participant baseline characteristics are presented in Table 1.

**Table 1.** Baseline Participant Characteristics

*Variable*	*Efficacy/safety population (*n* = 90)*
Age, years (SD)	45.1 (16.2)
Gender, *n* (%)	
Male	54 (60.0)
Female	36 (40.0)
Race, *n* (%)	
Caucasian	77 (85.6)
Black or African American	7 (7.8)
Asian	3 (3.3)
American Indian or Alaska Native	2 (2.2)
Native Hawaiian or Other Pacific Islander	1 (1.1)
BMI, kg/m^2^ (SD)	29.1 (6.2)
Years since diabetes diagnosis, years (SD)	20.1 (13.7)
Diabetes type, *n* (%)	
T1D	61 (67.8)
T2D	29 (32.2)
HbA1c, % (SD)	7.6 (1.2)
Diabetes therapy, *n* (%)	
Oral or diet and exercise^a^	22 (24.4)
Long acting insulin^b^	1 (1.1)
Multiple daily injections^c^	24 (26.7)
Insulin pump^d^	43 (47.8)
History of ketoacidosis, *n* (%)	0 (0)
History of severe hypoglycemia, *n* (%)	1 (1.1)

^a^ Participants with T2D.

^b^ Participant with T2D.

^c^ Includes four participants with T2D.

^d^ Includes two participants with T2D.

BMI, body mass index; HbA1c, hemoglobin A1c; SD, standard deviation; T1D, type 1 diabetes; T2D, type 2 diabetes.

**FIG. 3.**
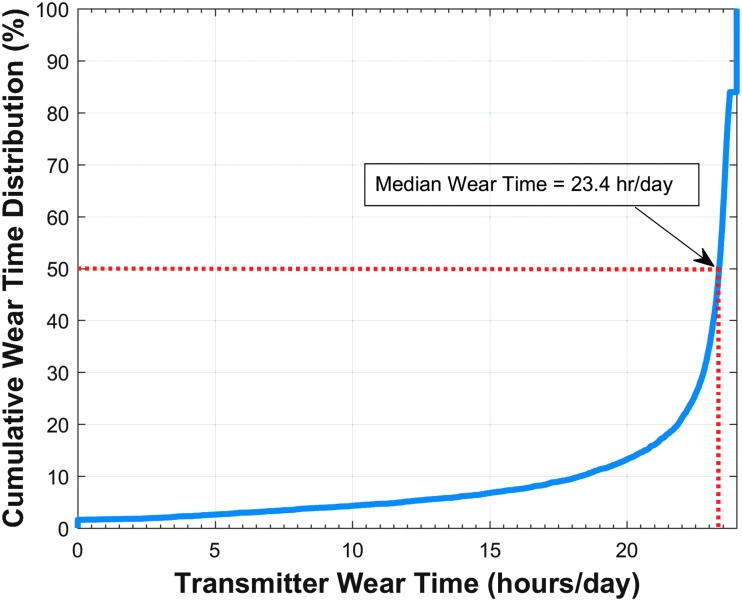
Cumulative frequency of transmitter use per day of all participants through 90 days or sensor retirement. Dotted red line shows that median wear time for the participants was 23.4 hours per day.

Figure 3 displays the cumulative frequency of transmitter use per day for all participants through sensor retirement or 90 days after sensor insertion. The frequency graph shows that the median transmitter wear time for the participants was 23.4 hours per day.

### Effectiveness outcomes

**FIG. 4.**
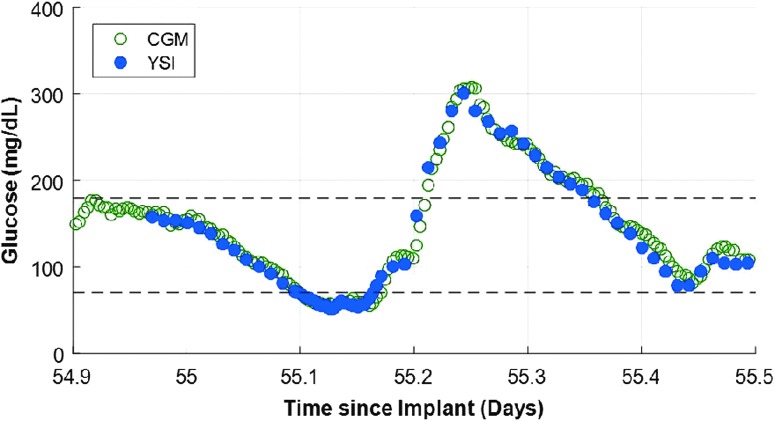
Example of an in-clinic session occurring on day 55 with YSI reference points illustrating the meal challenge excursions along with the continuous measurements from the sensor system. CGM, continuous glucose monitoring; YSI, Yellow Springs Instruments.

The primary effectiveness endpoint of MARD over the glucose range of 40–400 mg/dL was 8.8% (95% CI: 8.1%–9.3%) for the prespecified analysis population, which included the 82 participants who were not training participants and 16,653 matched glucose measurements (Table 2). Analysis showed that 93.3% of CGM values were within ±20 mg/dL or 20% of YSI reference values (referred to as 20/20%) over the total YSI glucose range of 40–400 mg/dL. The system showed accuracy and stability across all clinic visits with a high percentage of CGM values within 20/20% of reference values (Table 3). Similar results on the primary effectiveness endpoint were observed in the post hoc analysis of all 90 participants (18,261 matched glucose measurements) with an MARD of 8.9% (95% CI: 8.3%–9.4%); 93% of CGM values were within 20/20% of reference values. A representative example of an in-clinic session occurring with YSI reference points illustrating the meal challenge excursions along with the continuous measurements from the sensor system is presented in Figure 4.

**Table 2.** Continuous Glucose Monitoring System Accuracy and Stability Within Yellow Springs Instruments Glucose Ranges

*YSI glucose, range (mg/dL)*	*No. of paired CGM system-YSI reference readings*	*Percent within 15/15% reference*	*Percent within 20/20% reference*	*Percent within 30/30% reference*	*Percent within 40/40% reference*	*MARD (%) [95% CI]*
Overall	16,653	85.7	93.3	98.1	99.4	8.8 (8.1–9.3)
>40–54	167	83.2	85.6	93.4	96.4	10.7 (7.4–13.3)
55–70	785	86.1	92.9	96.8	98.9	9.0 (8.1–12.0)
71–180	8943	84.8	92.3	97.5	99.2	8.7 (8.2–9.5)
>180	6758	86.9	95.0	99.0	99.8	7.8 (7.3–8.8)

In-clinic accuracy is assessed compared with venous YSI reference measurement. The percentage of system readings within ±15 mg/dL or 15% of YSI reference values (15/15%), ±20 mg/dL or 20% of YSI reference values (20/20%), ±30 mg/dL or 30% of YSI reference values (30/30%), or ±40 mg/dL or 40% of YSI reference values (40/40%) are reported. For YSI ≤70 mg/dL, the differences in mg/dL are included instead of percentage difference (%).

CGM, continuous glucose monitoring; CI, confidence interval; MARD, mean absolute relative difference; YSI, Yellow Springs Instruments.

**Table 3.** Continuous Glucose Monitoring System Accuracy and Stability by Clinic Visit

*YSI glucose, range (mg/dL)*	*No. of paired CGM system-YSI reference readings*	*Percent within 15/15% reference*	*Percent within 20/20% reference*	*Percent within 30/30% reference*	*Percent within 40/40% reference*	*MARD (%) [95% CI]*
Day 1	1737	77.0	87.3	96.1	98.4	10.5 (9.4–11.6)
Day 30	5385	89.0	94.4	98.1	99.2	8.0 (6.8–8.8)
Day 60	5016	87.9	95.1	98.8	99.7	8.2 (7.3–8.9)
Day 90	4515	82.7	92.5	97.9	99.6	9.6 (8.6–10.5)

In-clinic accuracy is assessed compared with venous YSI reference measurement. The percentage of system readings within ±15 mg/dL or 15% of YSI reference values (15/15%), ±20 mg/dL or 20% of YSI reference values (20/20%), ±30 mg/dL or 30% of YSI reference values (30/30%), or ±40 mg/dL or 40% of YSI reference values (40/40%) are reported.

Table 4 provides the results on the effect of compression on accuracy. There was no significant difference in the percentage of CGM readings within 20/20% of the reference values for readings taken during compression (92.3%) or no compression (93.4%) conditions (*P* = 0.88). Table 5 provides the results on the effect of exercise on accuracy. No significant difference was observed in the percentage of CGM readings within 20/20% of the reference values between the exercise (95.1%) and nonexercise (93.2%) conditions (*P* = 0.35). Data obtained during the compression challenges did not indicate that the sensor signal was susceptible to the nighttime sensor attenuation phenomenon that is an issue with transcutaneous CGM sensors.^16,17^

**Table 4.** Continuous Glucose Monitoring System Accuracy During Compression Challenge (*n* = 15)

*Condition*	*No. of paired CGM system-YSI reference readings*	*Percent within 15/15% reference*	*Percent within 20/20% reference*	*Percent within 30/30% reference*	*Percent within 40/40% reference*	*MARD (%) [95% CI]*
Compression	274	86.1	92.3	96.0	99.6	9.3 (5.3–12.9)
No compression	16,379	85.7	93.4	98.1	99.4	8.7 (8.1–9.3)

In-clinic accuracy is assessed compared with venous YSI reference measurement. The effect of compression on accuracy was evaluated by instructing participants with bilaterally placed sensors to lie on one side for 30 min. Results compared with the noncompressed side are reported. The percentage of system readings within ±15 mg/dL or 15% of YSI reference values (15/15%), ±20 mg/dL or 20% of YSI reference values (20/20%), ±30 mg/dL or 30% of YSI reference values (30/30%), or ±40 mg/dL or 40% of YSI reference values (40/40%) are reported.

**Table 5.** Continuous Glucose Monitoring System Accuracy During Exercise Challenge (*n* = 70)

*Condition*	*No. of paired CGM system-YSI reference readings*	*Percent within 15/15% reference*	*Percent within 20/20% reference*	*Percent within 30/30% reference*	*Percent within 40/40% reference*	*MARD (%) [95% CI]*
Exercise condition	1190	87.6	95.1	99.7	99.9	8.3 (7.5–9.3)
Nonexercise condition	15,463	85.6	93.2	97.9	99.3	8.8 (8.1–9.3)

In-clinic accuracy is assessed compared with venous YSI reference measurement. The effect of exercise on accuracy was evaluated by instructing participants with a single sensor to perform upper arm exercises for 30 min. Results compared with a nonexercise condition are reported. The percentage of system readings within ±15 mg/dL or 15% of YSI reference values (15/15%), ±20 mg/dL or 20% of YSI reference values (20/20%), ±30 mg/dL or 30% of YSI reference values (30/30%), or ±40 mg/dL or 40% of YSI reference values (40/40%) are reported.

**FIG. 5.**
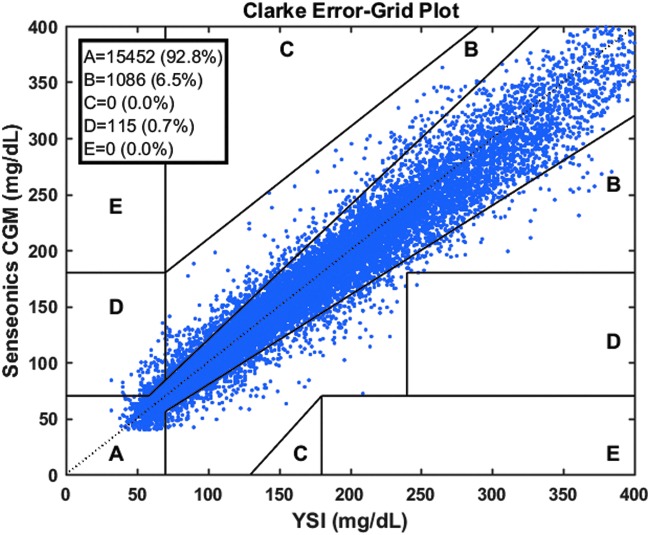
Clarke Error Grid analysis of CGM and YSI glucose measurements through 90 days.

The clinical performance of the CGM system estimated per Clarke Error Grid analysis showed 99.3% of samples in the clinically acceptable error zones A (92.8%) and B (6.5%), none in zones C and E, and 0.7% in zone D (Fig. 5).

Among the 15 participants who had bilateral sensor implantations, there were a total of 9974 matched pairs. Analysis demonstrated a strong correlation between sensors with a paired absolute relative difference of 8.8% (95% CI: 7.4%–12.3%).

The system remained accurate across all glucose ROC categories. The highest agreement with YSI glucose values was observed during the lowest CGM system absolute ROC (Table 6). CGM and YSI concordance by ROC is summarized in Table 7.

**Table 6.** Effect of System Rate of Change on Continuous Glucose Monitoring System and Reference Agreement

*CGM ROC [mg/(dL·min)]*	*No. of paired CGM system-YSI reference readings*	*Percent within 15/15% reference*	*Percent within 20/20% reference*	*Percent within 30/30% reference*	*Percent within 40/40% reference*	*MARD (%) [95% CI]*
Less than −2	251	80.9	90.8	95.6	98.4	10.5 (8.2–14.1)
−2 to −1	1285	82.8	91.7	96.9	98.7	10.1 (8.9–11.4)
−1 to 1	12,740	86.8	94.1	98.4	99.5	8.5 (7.7–8.8)
1 to 2	1188	80.6	89.7	97.8	99.7	9.4 (8.8–10.3)
>2	515	81.9	91.1	97.5	99.4	9.4 (8.3–10.2)

In-clinic accuracy is assessed compared with venous YSI reference measurement. The percentage of system readings within ±15 mg/dL or 15% of YSI reference values (15/15%), ±20 mg/dL or 20% of YSI reference values (20/20%), ±30 mg/dL or 30% of YSI reference values (30/30%), or ±40 mg/dL or 40% of YSI reference values (40/40%) are reported.

**Table 7.** Rate of Change Accuracy

	*YSI reference ROC [mg/(dL·min)]*					
	*Number and percent of matched pairs in each reference*					
	*Trend range for each CGM trend range*					
*CGM trend [mg/(dL·min)]*	*Less than −2*	*[−2, −1)*	*[−1, 1]*	*(1, 2]*	*>2*	*Total*
Less than −2	74	106	60	1	0	241
	30.7%	44.0%	24.9%	0.4%	0.0%	100.0%
[−2, −1)	53	448	745	7	3	1256
	4.2%	35.7%	59.3%	0.6%	0.2%	100.0%
[−1, 1]	51	541	11036	622	141	12391
	0.4%	4.4%	89.1%	5.0%	1.1%	100.0%
(1, 2]	3	10	600	414	129	1156
	0.3%	0.9%	51.9%	35.8%	11.2%	100.0%
>2	0	1	99	164	236	500
	0.0%	0.2%	19.8%	32.8%	47.2%	100.0%
Total	181	1106	12540	1208	509	15544

ROC, rate of change.

In cases wherein CGM and YSI were discordant, they were usually only one category away from the main diagonal. Only 2.4% (376 pairs) of the available pairs were more than one category away from the main diagonal for the system-reported ROC.

The ability of the system to identify hypoglycemic and hyperglycemic events showed a relatively high degree of performance. The system correctly identified 93% and 96% of hypoglycemic and hyperglycemic events per YSI. When a hypoglycemic or hyperglycemic event was detected by the device, the system determination was in agreement with YSI in 86% and 94% of cases, respectively (Table 8).

**Table 8.** In-Clinic Hypoglycemic and Hyperglycemic Event Detection Using Both Threshold and 10 Minutes Predictive Alerts

*Glucose setting (mg/dL)*	*Confirmed event detection rate*	*Missed event detection rate*	*True alert rate*	*False alert rate*
Hypoglycemic alert				
70	93%	7%	86%	14%
Hyperglycemic alert				
180	96%	4%	94%	6%

**FIG. 6.**
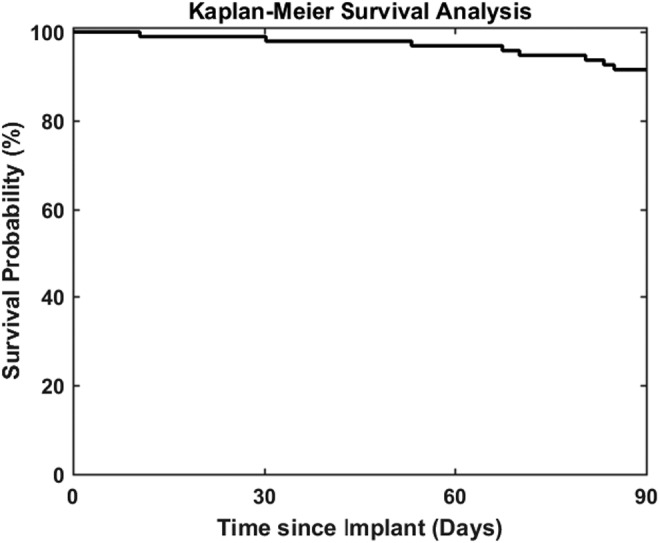
Kaplan–Meier analysis of sensor survival probability through 90 days.

The sensor survival probability at day 90 for the 106 implanted sensors was 91% based on Kaplan–Meier analysis (Fig. 6). For the sensors that retired before day 90, one of the sensors that triggered a retirement alert was due to a compromised electrical connection in the sensors optics. This sensor was replaced, per protocol, since it occurred within 30 days of insertion. All other early sensor retirements were due to the sensor reaching insufficient sensitivity to glucose from degradation of the binding chemistry as described by Colvin and Jiang.^20^

### Safety outcomes

During the study, there were a total of 14 adverse events among seven participants that were adjudicated as either related or possibly related to the device or insertion/removal procedures. Two events were classified as moderate in severity and 12 events were classified as mild. Nine events were bruising, erythema, or pain/discomfort; eight of these events were considered mild in severity and one instance of arm pain was of moderate severity. One syncopal episode after insertion of the device and one episode of paresthesia or tingling were reported: both were rated mild in severity. There were two events wherein it could not be assured that a small element of the PMMA encasement was removed (rated as mild in severity due to the small size of the element and biocompatibility of the material). There was also one event of an inability to remove the sensor on first attempt. The investigator referred the participant to a general surgeon who elected to use general anesthesia for the removal procedure, resulting in the event being adjudicated as serious. All events had completely resolved at the conclusion of the study with the exception of one participant who reported mild discomfort at the sensor site after removal of the sensor, which resolved with no intervention after database closure. There were no incisional infections at insertion or removal. In addition, no skin reactions due to the adhesive patch were observed. Lastly, plasma dexamethasone levels were undetectable (<2 ng/mL) for all participants before insertion and at day 90.

## Discussion

This 90-day, nonrandomized, blinded, prospective, single-arm multicenter study demonstrated both accuracy and safety of the implantable Eversense CGM system over the 90-day sensor life. The system was accurate, with an overall MARD of 8.8% across the clinically relevant glucose range (40–400 mg/dL) with 93.3% of CGM values within 20/20% of reference values. There was no impact from either exercise or compression on sensor recording or values. Moreover, the Clarke Error Grid analysis showed high clinical performance with 99.3% of samples in the clinically acceptable error zones A and B. As with other commercially available CGM systems,^22–25^ the sensor was less accurate in the hypoglycemic range (≤70 mg/dL).

The system had a favorable safety profile for its intended use. Clinicians with limited to no surgical experience were able to insert and remove the sensor without difficulty after appropriate training. The insertion, use, and removal of 106 glucose sensors in 90 participants resulted in few mild to moderate adverse events and one SAE. This event involved the unsuccessful removal attempt and the need to refer a participant to a surgical specialist for an outpatient procedure, who elected to use general anesthesia during the procedure, resulting from the sensor being placed too deeply at insertion. In addition, there were no detectable levels of dexamethasone in plasma.

The Eversense CGM system was designed, in part, to offer a treatment option that may better accommodate patients' preferences and lifestyles. The high rate of adherence in the study with a median device wear time of 23.4 hours per day supports that the system did not interfere with daily living. Furthermore, despite the extensive wear times, there were no adhesive patch skin reactions, which is of particular importance as skin reactions to adhesives continue to be relatively common with other CGM systems.^26,27^

The results of the PRECISE II study compare favorably with the original Eversense CGM system, which was evaluated in the PRECISE study.^18^ The new, updated Eversense system evaluated in PRECISE II relocated the dexamethasone ring closer to the optical detection zone of the sensor and updated the software algorithm. The MARD value against reference glucose values was lower in the PRECISE II study than what was observed in the PRECISE study (8.8% vs. 11.6%). The new sensor and algorithm configuration also appears to provide greater sensor longevity through 90 days (91% vs. 82%) and greater accuracy in terms of confirmed detection rates for hypoglycemic (93% vs. 81%) and hyperglycemic (96% vs. 88%) events.

Owing to the blinding of the real-time CGM display and the device alerts during the study, a key limitation of the study was the inability to assess the full utility of the device by the users. Even with this limitation, a 0.5 percentage point reduction in HbA1c from a baseline of 7.6% was observed at 90 days postinsertion (*P* < 0.0001). In addition, the recent study by Kropff et al. using the original system in a nonblinded manner also reported an association between CGM system use and a significant reduction in HbA1c, from 7.5% at baseline to 7.2% at study end, (*P* < 0.001).^18^ A quality-of-life substudy within PRECISE found that participants viewed the implantable CGM favorably with 86% of participants reporting feeling better about their diabetes control using the CGM Impact scale and 84% of participants reported that they would choose to have a sensor inserted again.^28^ An additional limitation was under-representation of non-Caucasian participants. Finally, although the study involved placement of a single sensor for 90 days, the expectation would be that patients have serial sensors placed over their lifetime. Long-term surveillance studies will be required to ensure that the safety profile remains favorable with multiple sensor placements and removals.

## Conclusions

The results from this study demonstrate that the use of a long-term, 90-day, implantable continuous glucose sensor is accurate and safe with high rates of adherence to use. Additional clinical studies will be required to evaluate the accuracy and usability of the Eversense CGM system among pediatrics, with reduced calibration frequency, and for extended durations through 180 days.
